# 多肽功能化亲和微球的制备与线粒体高选择性分离分析

**DOI:** 10.3724/SP.J.1123.2024.01013

**Published:** 2024-06-08

**Authors:** Jian CHEN, Kun XU, Han GAO, Rui ZHAO, Yanyan HUANG

**Affiliations:** 1.北京分子科学国家研究中心, 中国科学院化学研究所, 中国科学院活体分析化学重点实验室, 北京 100190; 1. Beijing National Laboratory for Molecular Sciences, CAS Key Laboratory of Analytical Chemistry for Living Biosystems, Institute of Chemistry, Chinese Academy of Sciences, Beijing 100190, China; 2.中国科学院大学, 北京 100049; 2. University of Chinese Academy of Sciences, Beijing 100049, China

**Keywords:** 多肽, 亲和识别, 分离材料, 线粒体, 复杂体系, 液相色谱-串联质谱, peptide, affinity recognition, separation materials, mitochondria, complicated systems, liquid chromatography-tandem mass spectrometry (LC-MS/MS)

## Abstract

线粒体作为细胞物质和能量代谢的主要细胞器,在维持细胞生理稳态中发挥关键作用,进而与诸多疾病的发生发展密切相关。从高度复杂的细胞组分中特异性分离分析线粒体,对于其功能解析、分子机制研究和化学干预具有重要意义,但仍存在困难与挑战。本研究以靶向多肽为识别元件,开展了线粒体亲和分离新材料的设计、合成和分析应用研究。以与线粒体膜具有特异性相互作用的线粒体穿透肽(mitochondrial penetrating peptide, MPP)为识别元件,通过引入空间手臂分子,设计合成了多肽亲和配基。以表面富含醛基的聚合物基质微球(matrix beads, MB)为亲和基质,建立了基于胺甲基化反应的多肽功能化亲和微球制备方法,该方法具有修饰反应条件温和、配基修饰效率高的特点。将所构建的亲和微球MB@MPP用于细胞破碎液中线粒体的直接分离,实现了完整线粒体的快速、高选择性和无损捕获。与商品化分离试剂盒相比,MB@MPP捕获的线粒体中标志蛋白富集效率更高,纯度更好。基于多肽功能化微球对线粒体高选择性分离能力,以线粒体代谢所需关键辅酶的前体小分子色氨酸和核黄素为目标,进一步建立了基于液相色谱-串联质谱的线粒体中活性小分子的分析检测方法,实现了激酶激动剂刺激前后线粒体中活性小分子含量及其动态变化分析,为线粒体功能解析和相关疾病分子机制研究提供了方法基础。

## Abstract (en)

Mitochondria perform various metabolic processes that significantly affect cell differentiation, proliferation, signal transduction, and programmed cell death. The disruption of mitochondrial bioenergetic and metabolic functions is closely related to many disorders. The specific isolation and purification of intact, high-purity, and functional mitochondria are central to the understanding of their mechanism of action but remain challenging tasks.

In this study, a mitochondrial penetrating peptide (MPP) with the sequence FrFKFrFK(Ac) was used as a mitochondrial recognition motif to construct a peptide-guided affinity separation material. The multiple aromatic phenylalanine (F) residues in this amphiphilic peptide can confer lipophilicity to the mitochondrial membrane, whereas the basic residues (D-arginine and lysine) render the MPP surface positively charged, thereby promoting the binding of negatively charged mitochondria. After the derivatization of the N terminal of MPP with an oligoglycine spacer, the peptide ligands were conjugated to matrix beads (MB) with surface aldehyde functional groups. Peptide functionalization was performed via a condensation reaction between the amino group in the peptide ligand and the aldehyde group on the beads. The generated Schiff bases were reduced, affording stable covalent bonds. The dense and stable functionalization of the beads with the mitochondria-targeting peptides was demonstrated using high performance liquid chromatography (HPLC), zeta potential assay, and scanning electron microscopy (SEM). The immobilization efficiency of the peptide ligands was 1.47 μmol/g, and the surface potential of MB@MPP was 11 mV. MB@MPP was used for the direct isolation of mitochondria after cell homogenization. As observed by SEM, mitochondria with a cross-sectional diameter of 500 nm were efficiently captured on the MB@MPP surface. Because the mitochondrial membrane potential is an important marker of mitochondrial function and the driving force behind the staining of mitochondria with Mito Tracker dyes, the specific binding and separation of fluorescent mitochondria from the cell samples revealed that the proposed MB@MPP-based isolation approach can keep mitochondria intact and retain their functions. Western blot assays were employed to characterize the protein markers of the mitochondria (citrate synthase (CS) and voltage-dependent anion channel protein (VDAC)) and cytoplasmic protein (vinculin), and examine the integrity and purity of the captured mitochondria. The results showed that the lysates released from MB@MPP had high CS and VDAC contents. By contrast, vinculin, which is highly abundant in whole-cell lysates, was barely detected in the lysates from MB@MPP. These results suggest that MB@MPP isolates mitochondria with high affinity, specificity, and antifouling ability by using the targeting peptide as the capture handle. A comparison with a commercial mitochondrial isolation kit demonstrated that MB@MPP can separate mitochondria with higher CS and VDAC abundance and purity. Given the superior separation performance of MB@MPP, the molecular profiles of the isolated mitochondria under stress were subjected to further analysis of their molecular profiles under stress.

A liquid chromatography-tandem mass spectrometry (LC-MS/MS) method was established to detect tryptophan (Trp) and riboflavin in the mitochondria. Quantification was performed in multiple-reaction monitoring (MRM) mode. Owing to the high purity of the mitochondria, the Trp and riboflavin contents were determined to be 265 and 0.67 nmol/mg, respectively. The metabolic response of mitochondria to external stimuli was further examined using acadesine, an adenosine 5'-monophosphate (AMP)-activated protein kinase activator with a wide range of metabolic effects, to treat cells. After cell homogenization, MB@MPP was used to separate the mitochondria from the cell samples with and without acadesine treatment, followed by LC-MS/MS analysis. The quantification results demonstrated that acadesine induced a 14% upregulation of Trp content in the mitochondria. By contrast, the riboflavin content decreased to 0.48 nmol/mg, which is 72% of that in untreated mitochondria. The changes in Trp and riboflavin contents could influence their metabolic pathways and, thus, the levels of their metabolites, such as nicotinamide adenine dinucleotide, flavin mononucleotide, and flavin adenine dinucleotide, which are essential coenzymes in mitochondria. Peptide-functionalized affinity microbeads with high affinity and specificity for mitochondria are promising for the efficient isolation of high-quality mitochondria, and offer a useful tool for understanding the complicated functions and dynamics of this unique organelle.

线粒体作为细胞生命活动的重要场所,拥有一套复杂而精细的分子相互作用网络和分子反应系统,不仅是细胞的能量工厂,还参与并调控细胞分化、细胞周期、信号传导和凋亡等重要生命过程^[1⇓-3]^。已有研究表明,线粒体的异常和功能障碍与衰老、神经退行性疾病、糖尿病、癌症等重大疾病密切相关^[4⇓-6]^。近年来,探测和调控线粒体及相关生命活动已成为化学和生物学关注的热点研究领域^[7⇓-9]^。其中,构建线粒体特异性分离材料,建立线粒体化学与生物信号分子的高灵敏测量新方法,是线粒体结构与功能研究亟需的关键环节,对于相关疾病靶标的发现和分子机制研究具有重要意义。

亚细胞组分分离技术的发展使线粒体及相关分子的靶向分析成为可能。目前,基于物理性质的超速离心、密度梯度离心等技术广泛用于线粒体分离之中,但仍存在产物纯度有限、易受细胞碎片等杂质干扰等问题^[10,11]^。近年来,电泳、场流分离等技术逐渐被用于线粒体分离分析之中,在分离速度和效率等方面获得了显著提升^[12,13]^。基于分子识别的亲和分离是高效、高选择性捕获富集线粒体的有效途径^[14]^。利用抗原-抗体的特异性相互作用,研究人员建立了基于亲和免疫沉淀技术的线粒体快速分离方法,结合高效液相色谱-质谱(HPLC-MS)联用,分离鉴定了传统方法未能鉴别的线粒体内小分子代谢物^[15,16]^。Chang等^[17]^将线粒体膜蛋白抗体修饰到聚多巴胺包覆的磁性氧化石墨烯上,制备了新型的线粒体免疫亲和分离材料,具有选择性好、稳定性高且易于磁分离的优点,实现了细胞和线粒体中多种代谢物的差异分析。Ahier等^[18]^将免疫识别与磁分离相结合,以特定细胞中线粒体标志蛋白为靶标,采用抗体修饰免疫磁珠为分离材料,建立了细胞中特异性线粒体的亲和纯化方法,实现了不同细胞谱系线粒体之间蛋白质和核酸组成的比较分析。随着线粒体结构与功能研究的日益深入,其选择性分离分析呈现出新需求和新挑战。作为半自主细胞器,线粒体不仅具有自身遗传物质和蛋白质表达系统,还时刻对细胞内外的生理病理信号和刺激产生响应,调整物质和能量代谢所需的众多生化反应。因此,为了解析高度复杂且动态变化的生命过程,高选择性分离获取高纯度、结构完整和功能无损的线粒体成为亟需解决的关键问题。

多肽作为内源性生物活性分子,在复杂生命体系的快速、可控、动态分析中表现出巨大的潜力^[19⇓-21]^。与抗体等生物大分子亲和配基相比,多肽具有分子尺寸小、构象灵活、膜穿透能力强和生物相容性好的特点;不仅如此,多肽结构可设计、可化学合成和修饰、功能可调控,是理想的靶向识别配体。利用多肽作为亲和配基制备分离材料,已成为蛋白质等生物大分子亲和纯化的有效途径之一^[22,23]^。近年来,多肽逐渐被用于特定细胞、细胞分泌囊泡的亲和捕获之中,实现了血液等复杂样品中循环肿瘤细胞、外泌体、蛋白聚集体等的靶向分离分析,为疾病诊疗、溯源和预后提供了重要信息^[24⇓⇓-27]^。

本研究针对线粒体特征的物理和化学性质,以特异性识别线粒体的两亲性且携带正电荷的线粒体穿透肽(mitochondrial penetrating peptide,MPP)为靶向配基,以富含醛基的乳胶为基质微球(matrix beads, MB),设计构建了线粒体亲和分离新材料;建立了细胞样品中线粒体的直接、快速、高选择性分离提取新方法,实现了完整线粒体的无损捕获。所发展的分离材料和方法不依赖特定蛋白靶标,在多肽识别导向下,所捕获的线粒体具有特征蛋白纯度高、富集效率好的特点。进一步发展了液相色谱-串联质谱(LC-MS/MS)方法,开展了线粒体中活性小分子色氨酸(Trp)和核黄素(riboflavin)的含量监测,实现了激酶激动剂刺激下线粒体内活性小分子的靶向分析,为线粒体功能解析和相关疾病分子机制研究提供了新方法。

## 1 实验部分

### 1.1 仪器、试剂与材料

LC-20AR半制备HPLC系统(岛津,日本); LC-20AD分析型HPLC系统(岛津,日本); UltiMate 3000型超高效液相色谱-电喷雾离子源-离子肼质谱分析系统(UHPLC-ESI-IT MS/MS, ThermoFisher,美国);配有基质辅助激光解吸电离源的9.4T Solarix型傅里叶变换离子回旋共振质谱仪系统(MALDI-FT ICR MS,布鲁克,德国); LC-MS 8040型高效液相色谱-三重四极杆质谱系统(岛津,日本); S-4800扫描电子显微镜(SEM, Hitachi,日本); Olympus FV 1000-IX81激光共聚焦显微镜(Olympus,日本); Zetasizer Nano ZS ZEN3600 Zeta电位分析仪(马尔文,英国)和5200 Multi化学发光成像系统(Tanon,中国)。

9-芴甲氧羰基(Fmoc)保护的氨基酸和1-羟基苯并三唑水合物(HOBt)购自Advanced ChemTech公司(美国); 2-(3'-*N*-氧代-苯并三唑)-1,1',3,3'-四甲基脲六氟磷酸盐(HBTU)和Fmoc-Rink Amide AM树脂(键合量为0.746 mmol/g)购自GL Biochem公司(上海);醛/硫酸盐乳胶微球、*N*,*N*-二甲基甲酰胺(DMF, HPLC级)、三异丙基硅烷(triisopropylsilane, TIS)、Mito Tracker Green、Dulbecco’s Modified Eagle’s Medium(DMEM)培养基和Micro BCA蛋白检测试剂盒购自Thermo Fisher Scientific公司(美国);色氨酸标准品(纯度99%)、核黄素标准品(纯度98%)、*N*-甲基吡咯烷酮、盐酸羟胺、三氟乙酸(TFA)、*N*-甲基吗啡啉购自百灵威科技有限公司(北京);阿卡地辛(acadesin,纯度99%)和甘氨酸(Gly,纯度99%)购自上海阿拉丁生化科技股份有限公司;线粒体分离试剂盒购自Sigma-Aldrich公司(美国); Radio Immunoprecipitation Assay(RIPA)裂解液、苯甲基磺酰氯(PMSF)、十二烷基硫酸钠-聚丙烯酰胺预制凝胶和蛋白酶抑制剂购自碧云天生物技术有限公司(上海); Vinculin一抗(ab129002, 1∶5000)、电压依赖性阴离子通道一抗(VDAC, ab14734, 1∶1000)、柠檬酸合酶(citrate synthase, CS)一抗(ab129095, 1∶1000)、辣根过氧化物酶(HRP)标记山羊抗兔IgG抗体(ab205718, 1∶5000)和HRP标记山羊抗鼠IgG抗体(ab205719, 1∶5000)购自Abcam公司(英国);人肝癌细胞HepG2购自中国科学院上海生命科学研究院(上海)。

### 1.2 MPP的固相合成、分离纯化与鉴定

采用Fmoc固相多肽合成法制备寡聚甘氨酸衍生的线粒体靶向多肽,具体步骤可参考文献[28]。以Fmoc-Rink Amide AM树脂作为起始固相载体,使用含有20%(v/v)六氢吡啶的DMF溶液为Fmoc脱保护剂。将物质的量为树脂负载氨基酸理论值4倍的Fmoc-氨基酸、HBTU和HOBt共同溶解在含有0.4 mol/L *N*-甲基吗啡啉的DMF溶液中活化氨基酸,活化后的氨基酸溶液加入到树脂中进行偶联。重复脱保护和偶联步骤,完成所有氨基酸的偶联。使用盐酸羟胺(1.80 mmol)和咪唑(1.35 mmol)的混合溶液(溶剂为5 mL *N*-甲基吡咯烷酮和1 mL二氯甲烷)作为C端赖氨酸侧链Dde保护基的脱除试剂。加入乙酸酐/吡啶/DMF混合溶液(1∶1∶8, v/v/v),将C端赖氨酸侧链氨基乙酰化。配制预冷的裂解液(TFA、水和TIS的体积比为95∶2.5∶2.5),将多肽从固相树脂中裂解出来。收集裂解液,通过旋转蒸发仪除去大部分TFA,并加入预冷的乙醚沉淀多肽,离心、真空干燥后得到多肽粗品,其氨基酸序列为NH_2_-Gly-Gly-Gly-Phe-(D)Arg-Phe-Lys-Phe-(D)Arg-Phe-Lys(Ac)-CONH_2_。

使用LC-20AR半制备型HPLC系统,对多肽粗品进行分离纯化,收集目标多肽的馏分,冷冻干燥后得到多肽纯品。使用LC-20AD分析型HPLC系统对纯化后的多肽进行分析,色谱柱为Halo Peptide ES-C18(150 mm×2.1 mm, 2 μm),流动相A为含有0.1%(v/v)TFA的水溶液,流动相B为含0.1%(v/v)TFA的乙腈溶液,流速为0.2 mL/min,在0~15 min内,梯度为5%B~60%B。利用MALDI-FT ICR MS和ESI-IT MS/MS获得目标多肽的精确相对分子质量及二级碎片信号。MALDI-FT ICR MS的扫描模式为正离子模式,基质为*α*-氰基-4-羟基肉桂酸,目标多肽[M+H]^+^理论值:*m/z* 1387.7877, [M+H]^+^实测值:*m/z* 1387.7855。

### 1.3 多肽功能化微球的合成与表征

取5 mg醛/硫酸盐乳胶微球,加入500 μL 0.4 mg/mL MPP水溶液,混合均匀后加入10倍量的氰基硼氢化钠作为还原剂,室温下反应12 h,通过多肽N端氨基与微球上醛基发生胺甲基化反应,将多肽键合至微球表面。为封闭微球表面剩余醛基及非特异性结合位点,加入500 μL 1 mol/L甘氨酸水溶液,室温下反应2 h,并用超纯水清洗3次,得到Gly封闭的MB@MPP,保存于超纯水中,用于后续线粒体的捕获。

配制质量浓度分别为0.01、0.025、0.05、0.1、0.25、0.4 mg/mL的MPP水溶液,使用分析型HPLC测定不同浓度下多肽的色谱峰面积,建立多肽峰面积与浓度的标准曲线。收集反应起始溶液、反应结束后上清液及清洗液进行HPLC分析,通过标准曲线计算单位质量微球上多肽的修饰量。

### 1.4 多肽功能化微球用于细胞样品中线粒体捕获

HepG2细胞使用添加有10%胎牛血清和1%双抗的DMEM高糖培养基培养。为考察药物刺激对线粒体代谢的影响,使用含有0.5 mmol/L阿卡地辛的细胞培养液进行培养,8 h后收集细胞,进行线粒体的分离分析。细胞生长至70%~80%汇合度后,使用KPBS(10 mmol/L KH_2_PO_4_, 136 mmol/L KCl, pH=7.25)清洗细胞,并用细胞刮刀收集细胞,离心、收集细胞沉淀。在沉淀中加入1 mL KPBS,混合均匀后使用玻璃匀浆器破碎细胞,并将匀浆液转移至离心管中,在1000 g下离心2 min,去除细胞碎片,上清液即为细胞破碎液。取200 μL细胞破碎液,加入1 mg MB@MPP, 4 ℃孵育2 h。离心分离微球(1000 g, 3 min),加入KPBS清洗微球1次,离心收集捕获线粒体后的微球,用于后续分析。

为了直接观测微球上捕获的线粒体,向细胞破碎液中加入线粒体特异性荧光染料Mito Tracker Green(200 nmol/L),在4 ℃下孵育20 min,对线粒体进行荧光标记。经离心清洗步骤,去除多余染料。按照上述步骤对细胞破碎液中荧光标记线粒体进行分离,使用FV 1000-IX81激光共聚焦显微镜进行成像分析,激发波长为488 nm, Mito Tracker Green染料的接收通道为500~600 nm。

### 1.5 蛋白质免疫印迹分析

向捕获有线粒体的MB@MPP中加入含有1%(v/v)PMSF和1%(v/v)蛋白酶抑制剂的RIPA裂解液,在冰上裂解15 min后,离心收集上清液,通过BCA蛋白定量法对上清液中蛋白质浓度进行测定。使用十二烷基硫酸钠-聚丙烯酰胺凝胶对上清液中的蛋白质进行电泳分离,总蛋白上样量为4 μg。取出预制胶,将蛋白质转印至聚偏二氟乙烯膜上,加入含5%脱脂奶粉的TBST溶液封闭1 h。加入1%脱脂奶粉稀释的一抗溶液,在4 ℃下孵育过夜。TBST清洗后,加入HRP标记二抗,室温下孵育1 h。TBST清洗后,向膜表面滴加化学发光检测液,使用5200 Multi化学发光成像系统进行显影,通过Image-J软件对蛋白条带灰度值进行定量分析。

### 1.6 捕获线粒体中活性分子的LC-MS/MS分析

#### 1.6.1 分析条件

称取Trp和核黄素标准品并溶解于80%(v/v)甲醇水溶液中,逐级稀释配制浓度分别为10、50、100、200、500、1000、2500和5000 nmol/L的标准溶液。使用LC-MS 8040系统对两种小分子进行分析,色谱柱为迪马Bio-Bond C8 300 Å(150 mm×2.1 mm, 3 μm),流动相A为0.1%(v/v)甲酸水溶液,流动相B为含0.1%(v/v)甲酸的乙腈溶液,流速为0.2 mL/min,在0~15 min内,梯度为20%B~60%B。

ESI离子源扫描模式为正离子模式,检测模式为多反应监测(MRM)模式,喷雾电压为5 kV,脱溶剂温度为280 ℃,喷雾气氮气流速为3 L/min,去溶剂气氮气流速为15 L/min,碰撞气压力为17 kPa,两种化合物的保留时间、一级母离子、二级碎片离子和碰撞能量见表1。对所选定碎片离子的峰面积进行积分,分别建立Trp和核黄素的峰面积与浓度标准曲线用于定量分析。

**表 1:** Trp和核黄素的保留时间和质谱参数

Compound	Retention time/min	Precursor ion (m/z)	Product ion (m/z)	CE/eV
Tryptophan	2.74	205.0	188.1^*^	10
			146.1	19
			118.1	26
Riboflavin	2.79	377.0	243.0^*^	24
			198.1	40
			172.7	40

* Quantitative ion. CE: collision energy.

#### 1.6.2 线粒体中小分子定量分析

按照1.4节步骤分离阿卡地辛给药组与未给药组细胞的线粒体,加入100 μL 80%(v/v)甲醇水溶液,剧烈涡旋10 min以提取线粒体内小分子。将线粒体提取液在14000 g下离心10 min,收集上清液并测定总蛋白浓度,使用LC-MS 8040系统对Trp和核黄素进行分析,通过标准曲线计算细胞和线粒体中小分子化合物的浓度,并将其换算为每毫克蛋白质中的相对浓度。最终实验结果为计算3次平行实验中小分子化合物相对浓度的平均值。

## 2 结果与讨论

### 2.1 多肽功能化线粒体亲和微球的设计

作为半自主细胞器,线粒体具有独特的物理化学性质,包括双层膜结构和膜两侧质子梯度所产生的负膜电位等,可为复杂体系中亲和分子探针的线粒体识别和定位提供重要依据。根据线粒体电子传输链电化学梯度导致的负膜电位(150~180 mV),线粒体穿透肽MPP(FrFKFrFK(Ac))同时含有芳香环和阳离子侧链,不仅携带正电荷,而且具有亲脂性,因此是理想的线粒体靶向结合配基^[29]^。作为基质材料,醛/硫酸盐乳胶微球MB粒径均匀,其表面富含的醛基为亲和配基的修饰提供了温和、易反应的位点。不仅如此,微球上高密度的硫酸盐极性基团,提供了亲水表面,并可在多肽共价修饰反应中有助于保持微球的稳定性。基于此,构建以MB为基质、线粒体靶向短肽MPP为表面识别元件的亲和分离材料MB@MPP,可望实现复杂样品中线粒体的高效、高选择性捕获。

亲和捕获微球MB@MPP的构建和线粒体分离分析示意图如图1所示。为了给亲和配基与线粒体的识别和相互作用提供充分的自由度,在靶向多肽MPP的N端引入寡聚甘氨酸(GGG)作为空间连接分子。利用微球表面醛基与多肽氨基之间的胺甲基化缩合反应,实现在温和条件下多肽配基的共价功能化。针对微球表面可能存在的未反应醛基,采用小分子甘氨酸为封闭试剂,可有效避免未反应醛基所引起的非特异性吸附,使MB@MPP同时具有线粒体专一性结合和抗干扰能力。

**图 1:**
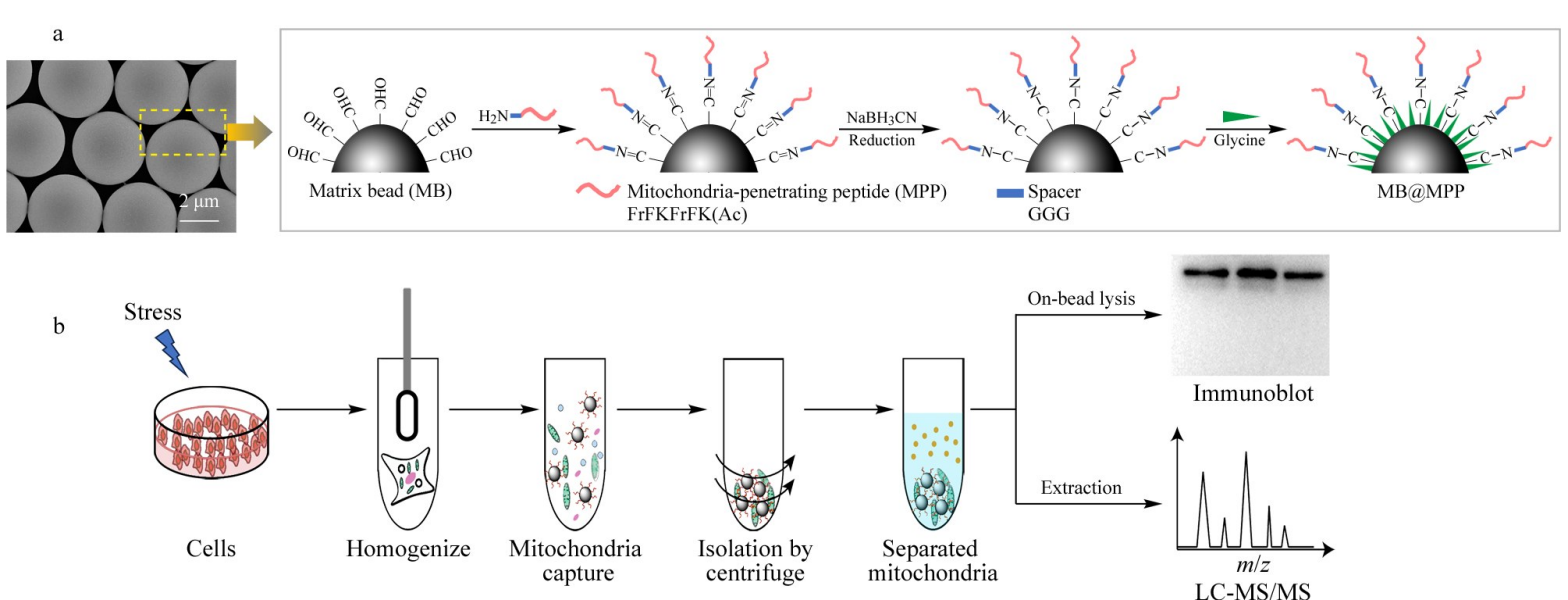
(a)多肽功能化亲和微球的构建示意图及其(b)用于线粒体靶向分离分析流程图

### 2.2 多肽功能化微球的制备与表征

采用Fmoc固相多肽合成法进行了寡聚甘氨酸手臂修饰的MPP亲和肽的制备。利用半制备高效液相色谱对多肽粗品进行分离纯化。HPLC和质谱分析结果表明,纯化后的亲和肽纯度高、结构正确(图2a,高分辨质谱结果见1.2节)。在MS/MS分析中,选取*m/z* 695.0的[M+2H]^2+^为母离子进行碎裂,检测到序列中不同位点化学键断裂后的二级碎片离子(图2b),进一步证明成功合成得到带有寡聚甘氨酸手臂的线粒体靶向多肽。

**图 2:**
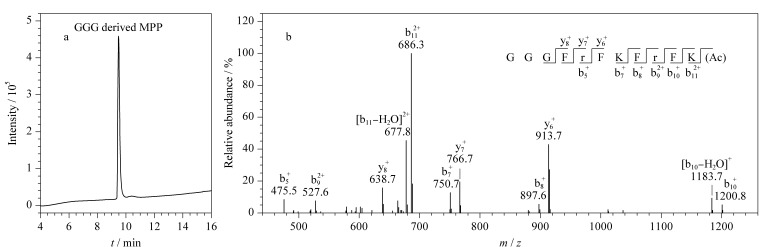
寡聚甘氨酸手臂衍生后的线粒体靶向多肽MPP的(a)HPLC图和(b)二级质谱图

在温和的水相体系中,开展了多肽对微球的功能化修饰反应。多肽氨基与微球醛基所形成的席夫碱,通过加入氰基硼氢化钠将其还原,从而形成稳定的共价偶联产物。利用HPLC检测亲和多肽的修饰效率,如图3a所示,反应12 h后,多肽的色谱峰面积明显下降,结合标准曲线进行定量计算,得到微球上多肽的键合量为1.47 μmol/g,该亲和配基的修饰密度可以满足亲和捕获的需要^[30⇓-32]^。

**图 3:**
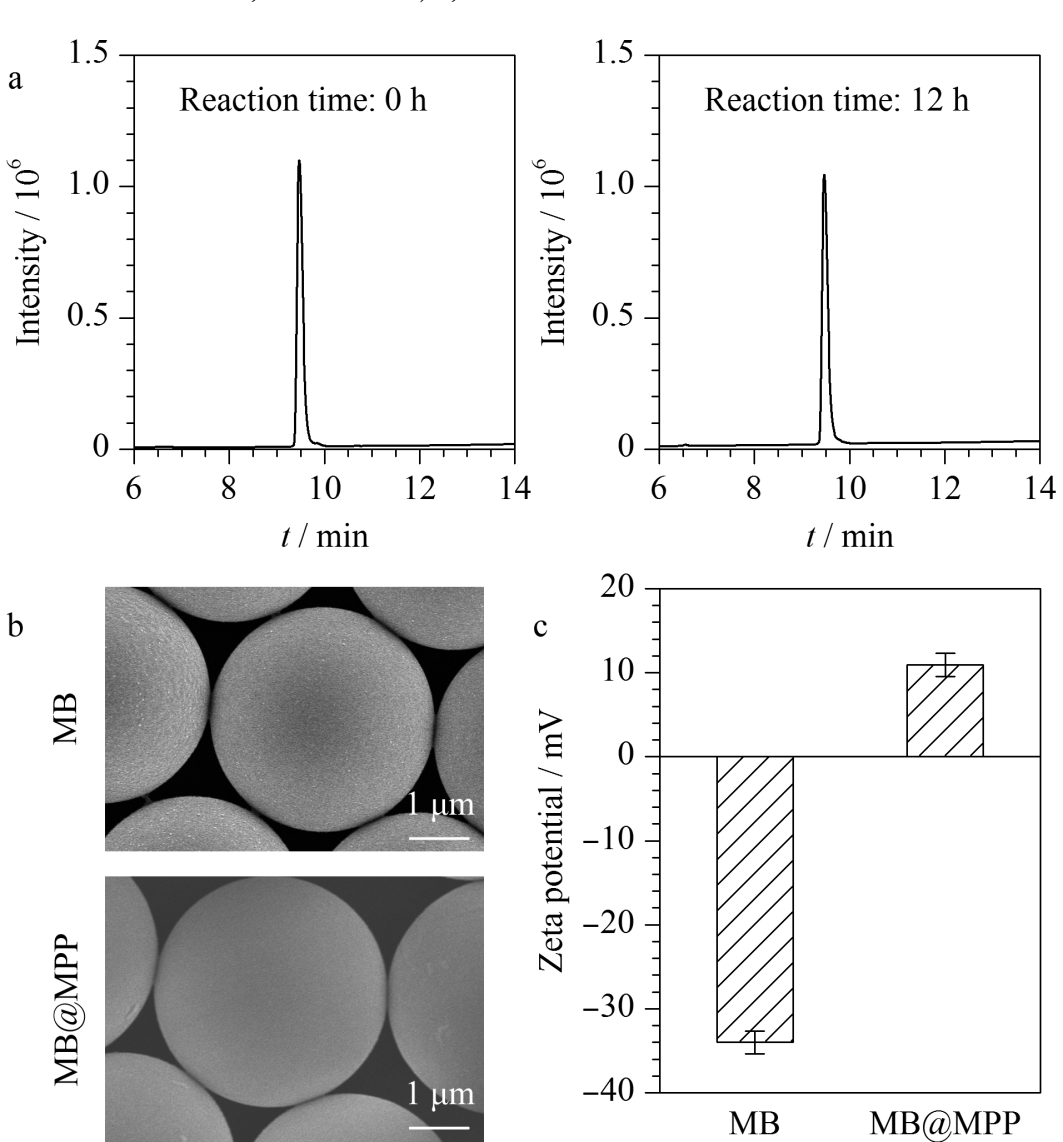
(a)RP-HPLC表征线粒体靶向多肽配基在MB微球上的修饰量,线粒体靶向多肽修饰前后微球的(b)SEM表征和(c)表面电荷表征(*n*=3)

利用SEM表征微球在亲和配基修饰前后的形貌,如图3b所示,得益于温和的修饰反应条件,微球尺寸和表面形貌未见改变。多肽修饰后的MB@MPP表面平整,可为线粒体的亲和捕获分离提供充分可及的结合位点。经Zeta电位表征,所购买的空白MB表面电荷为-34 mV,与其含有高密度的硫酸盐基团的化学组成相符。相比之下,多肽功能化后,MB@MPP微球表面电荷变为11 mV(图3c)。微球表面电荷的反转现象源于靶向多肽MPP中多个携带正电荷侧链的氨基酸残基(精氨酸和赖氨酸),不仅进一步证明多肽的高效修饰,而且MPP在生理条件下携带的正电荷通过静电相互作用与呈负电位的线粒体膜结合,有利于线粒体的亲和捕获。

### 2.3 细胞样品中线粒体的高选择性捕获

商品化线粒体提取试剂盒以及一些常规方法往往需要使用和LC-MS不兼容的提取试剂(含有高浓度溶质分子,如蔗糖),制约了后续线粒体成分和功能的研究^[15]^。以所构建的多肽功能化微球为亲和分离材料,建立了细胞裂解液中线粒体的直接快速分离方法,可以在LC-MS兼容的KPBS缓冲液中进行线粒体的特异性捕获。以肝癌细胞HepG2为模型,将其机械破碎后,直接加入MB@MPP微球进行靶标细胞器的识别与结合。利用SEM表征与细胞破碎液孵育后的MB@MPP,如图4a所示,观测到微球表面结合大量横截面直径为500 nm左右、形貌完整、呈短棒状或线状的线粒体;同时,微球表面未见其他微纳尺寸的细胞器吸附,表明多肽功能化微球可从复杂的细胞裂解样品中高选择性地识别并捕获线粒体,且分离得到的线粒体结构完整无损。进一步采用对细胞样品中的线粒体预先荧光染色的方法,考察MB@MPP对线粒体的捕获效率。通过共聚焦激光扫描显微镜观察,视野中的所有微球表面均呈现密集明亮、线粒体发出的绿色荧光(图4b),表明在多肽靶向识别作用下,MB@MPP可从细胞样品中高效分离富集线粒体。由于荧光染料与线粒体的特异性结合依赖于线粒体活性相关的跨膜电位,因此微球上仍发射荧光的线粒体说明所捕获的线粒体完好地保持了天然活性。

**图 4:**
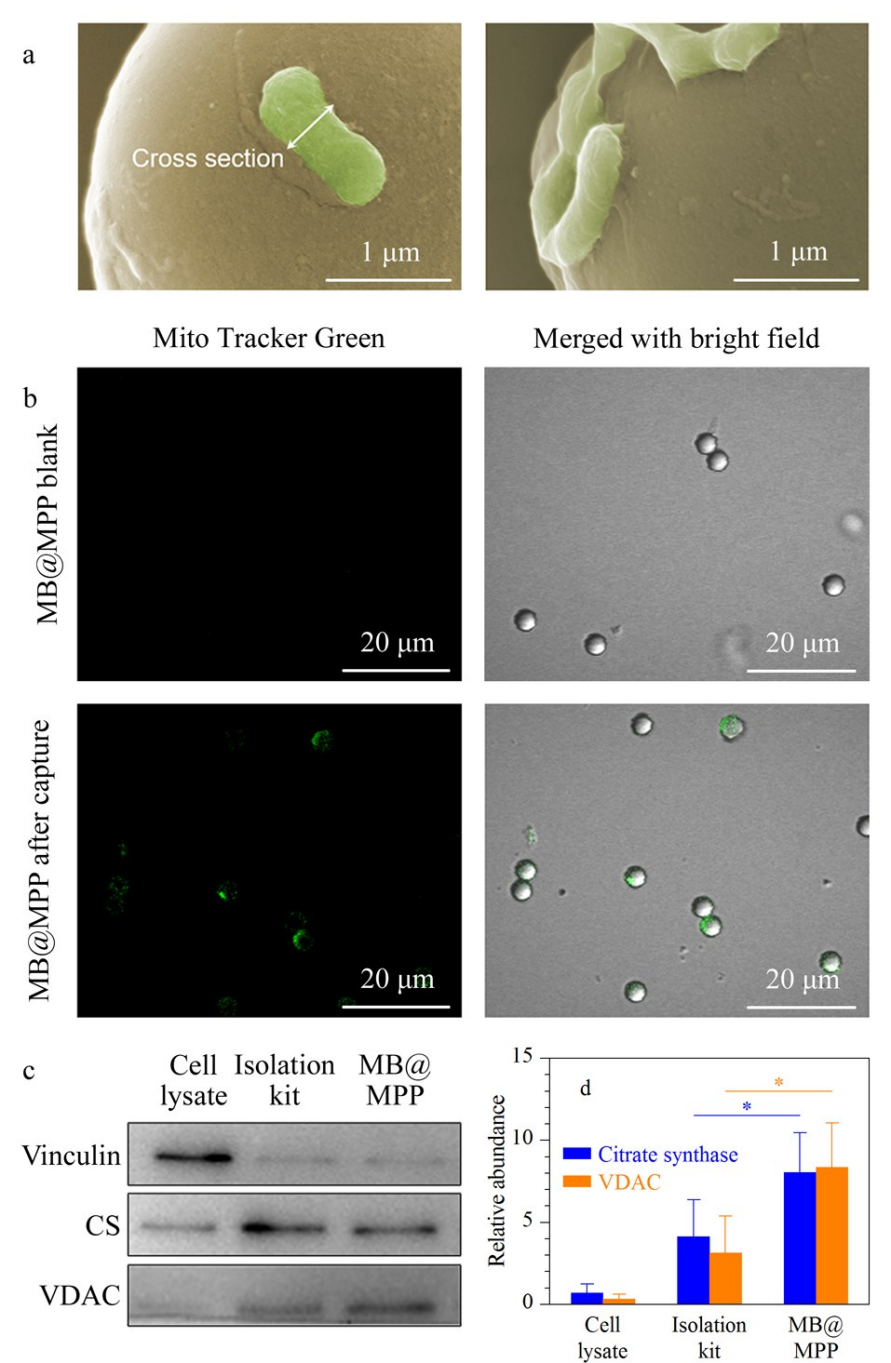
(a)SEM和(b)共聚焦激光扫描显微镜表征MB@MPP 对线粒体的特异性捕获能力,(c)免疫印迹法表征细胞破碎液、商品化试剂盒提取线粒体和MB@MPP捕获线粒体中的蛋白,(d)根据图 4c中的免疫印迹结果,计算细胞破碎液、商品化试剂盒提取线粒体和MB@MPP捕获线粒体中线粒体标志蛋白的相对含量

为了考察多肽功能化微球捕获线粒体的纯度和完整性,利用免疫印迹法(Western Blot)对线粒体标志蛋白CS、VDAC及细胞质蛋白Vinculin进行相对定量分析。CS是三羧酸循环中的关键酶分子,存在于线粒体基质之中;VDAC则调控线粒体内外物质交换,嵌入线粒体外膜;二者在线粒体代谢中发挥重要作用。如图4c所示,MB@MPP分离得到的产物中检测到明显的CS与VDAC条带,其强度显著高于全细胞裂解液,进一步证明材料具有线粒体捕获富集能力。对于全细胞裂解液中大量表达的细胞骨架蛋白兼黏着斑组成蛋白Vinculin, MB@MPP捕获产物中含量极少,表明微球具有抗污能力、非特异性吸附低。将多肽功能化微球与商品化线粒体提取试剂盒进行比较,对于同样的细胞样品,二者对细胞质蛋白Vinculin吸附极低,显示出高选择性(图4c)。进一步根据免疫印迹结果,计算相同总蛋白浓度下,捕获产物中线粒体标志蛋白CS和VDAC的相对含量。如图4d所示,MB@MPP捕获产物中CS和VDAC的相对含量显著高于试剂盒提取产物,显示多肽功能化微球在多肽分子识别下对线粒体具有高效捕获和富集能力。

### 2.4 线粒体内活性小分子分离分析

线粒体膜和基质中存在氧化磷酸化呼吸链、三羧酸循环等大量时刻发生生化反应的分子体系,是线粒体和细胞功能得以维持的基础。监测和分析生理病理条件下线粒体的生化反应和小分子物质,对于生命过程的认识具有重要意义。Trp和核黄素是线粒体代谢反应所需关键辅酶的前体^[33,34]^,被细胞摄取后分别转化为烟酰胺腺嘌呤二核苷酸(nicotinamide adenine dinucleotide, NAD)和黄素单核苷酸(flavin mononucleotide, FMN)、黄素腺嘌呤二核苷酸(flavin adenine dinucleotide, FAD),参与线粒体氧化呼吸链,是三羧酸循环的重要辅助因子^[35]^。基于此,利用多肽功能化微球对线粒体的高选择性、高效和无损分离能力,开展线粒体中Trp和核黄素的定量分析。采用二者的标准物质,通过仪器参数化和条件优化,建立了LC-MS/MS方法,通过MRM方式进行定量检测(图5a和b)。以Trp和核黄素的浓度为横坐标(*X*, nmol/L), MRM方法测定的提取离子流图峰面积为纵坐标(*Y*),拟合Trp的标准曲线线性方程为*Y*=1949.7*X*+45375,相关系数(*R*^2^)=0.9989;核黄素的标准曲线线性方程为*Y*=6629.1*X*+30096, *R*^2^=0.9967。利用所建方法,对MB@MPP捕获线粒体中的活性小分子进行分析。通过对定量碎片离子的峰面积进行积分,计算得到HepG2细胞线粒体中Trp和核黄素的含量分别为265 nmol/mg和0.67 nmol/mg。

**图 5:**
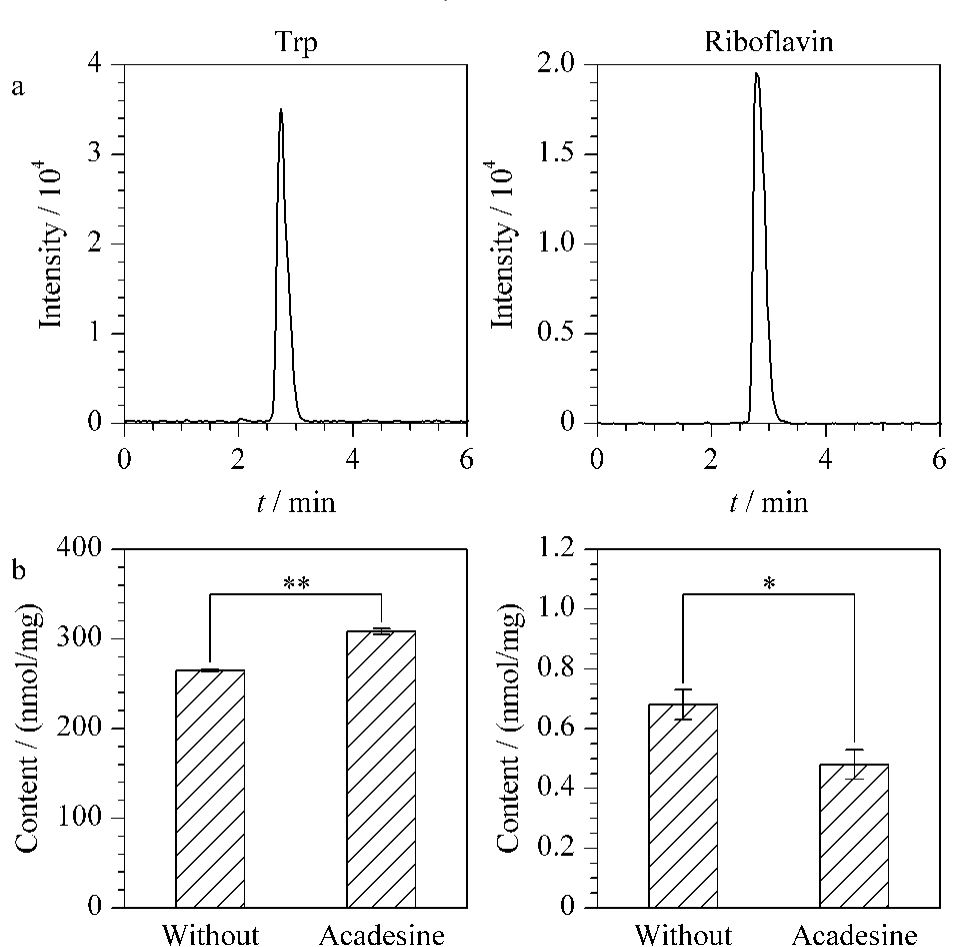
(a)Trp和核黄素的MRM色谱图,(b)药物分子 阿卡地辛处理前后,HepG2细胞的线粒体中Trp和核黄素含量变化

实验开展了药物刺激下,线粒体中活性小分子含量变化的分析。阿卡地辛作为一种腺苷类似物具有广泛的代谢调节作用,通过激活5'-腺嘌呤核苷酸(adenosine 5'-monophosphate, AMP)激活激酶,促进葡萄糖摄取、脂肪酸氧化和线粒体生物合成^[36]^,近年来被用于线粒体疾病治疗的研究之中^[37]^。基于此,将阿卡地辛作为药物刺激,与HepG2细胞共培养;利用MB@MPP分离药物刺激前后细胞内的线粒体;结合所建LC-MS/MS方法,对线粒体内Trp和核黄素含量进行检测。如图5b所示,阿卡地辛刺激后,线粒体中两种活性小分子的含量均产生显著变化。不同的是,阿卡地辛引起Trp含量上升了14%;而对于线粒体内的核黄素,阿卡地辛则导致其含量下降了28%(图5b)。上述实验结果提示,阿卡地辛作为激酶激动剂,对线粒体内Trp和核黄素的代谢具有调控作用,这可能与Trp和核黄素向NAD、FMN、FAD等辅酶转化过程中涉及磷酸化/去磷酸化反应过程相关。利用多肽功能化线粒体分离材料和液相色谱-质谱联用技术,可望为线粒体功能和小分子药物作用机制研究提供新方法。

## 3 结论

本研究以线粒体穿透肽为识别元件,设计合成了富含正电荷、两亲性且识别位阻小的多肽亲和配基。利用温和高效的共价修饰反应,构建了多肽功能化亲和微球,建立了复杂细胞样品中线粒体直接分离富集方法,具有捕获效率高、特异性好,分离得到的线粒体形貌完整、无损等特点。基于多肽功能化亲和材料良好的线粒体分离性能,建立了基于LC-MS/MS的Trp、核黄素定量检测方法,实现了激酶激活剂刺激前后线粒体中两种活性小分子含量的变化监测分析。所构建的线粒体捕获材料和靶向分析方法在线粒体功能和相关疾病分子机制研究中具有应用价值。
